# Use of Novel Concussion Protocol With Infralow Frequency Neuromodulation Demonstrates Significant Treatment Response in Patients With Persistent Postconcussion Symptoms, a Retrospective Study

**DOI:** 10.3389/fnhum.2022.894758

**Published:** 2022-05-24

**Authors:** Stella B. Legarda, Caroline E. Lahti, Dana McDermott, Andreas Michas-Martin

**Affiliations:** Neurology, Montage Health, Montage Medical Group, Monterey, CA, United States

**Keywords:** endogenous neuromodulation, infralow frequency brain training, concussion treatment, postconcussion syndrome, postconcussion symptoms, concussion protocol

## Abstract

**Introduction:**

Concussion is a growing public health concern. No uniformly established therapy exists; neurofeedback studies report treatment value. We use infralow frequency neuromodulation (ILF) to remediate disabling neurological symptoms caused by traumatic brain injury (TBI) and noted improved outcomes with a novel concussion protocol. Postconcussion symptoms (PCS) and persistent postconcussion symptoms (PPCS; >3 months post head injury) are designated timelines for protracted neurological complaints following TBI. We performed a retrospective study to explore effectiveness of ILF in PCS/PPCS and investigated the value of using this concussion protocol.

**Method:**

Patients with PCS/PPCS seen for their first neurology office visit or received their first neurofeedback session between 1 August 2018 and 31 January 2021 were entered. Outcomes were compared following treatment as usual (TAU) vs. TAU with ILF neurotherapy (TAU+ILF). The study cohort was limited to PPCS patients; the TAU+ILF group was restricted further to PPCS patients receiving at least 10 neurotherapy sessions. Within the TAU+ILF group, comparisons were made between those who trained at least 10 sessions using concussion protocol (TAU+ILF+CP) and those who trained for at least 10 sessions of ILF regardless of protocol (TAU+ILF-CP).

**Results:**

Among our resultant PPCS cohort (*n* = 59) leading persistent neurological complaints were headache (67.8%), memory impairment (57.6%), and brain fog (50.8%). PPCS patients in TAU+ILF+CP (*n* = 25) demonstrated greater net (*p* = 0.004) and percent (*p* = 0.026) improvement of symptoms compared to PPCS subjects in TAU (*n* = 26). PPCS patients in TAU+ILF-CP (*n* = 8) trended toward significant symptom improvements compared to TAU, and TAU+ILF+CP trended toward greater efficacy than TAU+ILF-CP.

**Conclusion:**

PPCS patients who received TAU+ILF+CP demonstrated significantly greater improvement as a group when compared to TAU. When used as an integrative modality to treatment as usual in managing patients with PPCS, ILF neuromodulation with use of concussion protocol provided significant symptom improvements.

## Background

Traumatic brain injury (TBI) is a major public health concern labeled a “silent epidemic” to characterize its true incidence as many cases go unrecognized and are excluded from official statistics (Rusnak, 2013). Traumatic brain injury has been estimated to affect 1.7 million people annually in the United States (Coronado et al., 2011), with a daily average of over 600 TBI-related hospitalizations and 176 TBI-related deaths (Centers for Disease Control and Prevention [CDC], 2022). The Centers for Disease Control (CDC) emphasize that the many TBIs treated in emergency rooms, urgent care and primary care settings or those who do not seek treatment are not included in these estimates (Bell et al., 2017). About 75% of TBIs are characterized as mild (mTBI), however, the true incidence of mTBI is very likely much higher given the foregoing observations; the CDC estimates that there are as many as 4 million mTBI cases annually in the United States alone. Worldwide the annual incidence of mTBI is estimated at 42 million which are most often due to falls and motor vehicle collisions (Cassidy et al., 2004).

There are no accurate estimates of the incidence and prevalence of “concussion” for these reasons. Concussion is increasing as a major public health concern because of its potential long-term effects, medically termed “postconcussion syndrome,” wherein somatic, cognitive, emotional, and/or behavioral complaints persist long after injury (Polinder et al., 2018). As these symptoms are not specific to TBI, the term “postconcussion symptoms” (PCS) has become the preferred, broadly inclusive diagnosis for the array of complaints sustained after TBI (Ng et al., 2019). Significantly, recent large epidemiological studies identify mTBI as a risk factor for frontotemporal dementia (Rosso et al., 2003; Kalkonde et al., 2012). Following significant biomechanical forces on the brain, TBI is often considered in three main categories: closed head injury, penetrating injury and blast injury.

### Clinical Mild Traumatic Brain Injury

The American Congress of Rehabilitation Medicine established criteria for defining mild traumatic brain injury (mTBI, see Table 1; Kay et al., 1993). Acute PCS, especially after mTBI, typically resolves within a few days to a few weeks without need for clinical intervention; however, in some cases symptoms may persist for months or even years without improvement (Dwyer and Katz, 2018). “Persistent post-concussion symptoms” (PPCS) has been temporally assigned to symptoms persisting beyond three months following a TBI (Bigler, 2008).

**TABLE 1:** Definition of mild traumatic brain injury (Kay et al., 1993; Varriano et al., 2018) (American Congress of Rehabilitation Medicine).

Traumatically induced physiological disruption of brain function as manifested
by at least one of the following:
– Any period of loss of consciousness
– Any loss of memory for events immediately before or after the accident
– Any alteration in mental state at time of accident
(e.g., dazed, disoriented, confused)
– Focal neurological deficit (s) that may/may not be transient and:
Loss of consciousness <30 min or GCS 13–15 if >30 min
posttraumatic amnesia <24 h

A number of premorbid conditions are largely agreed upon as risk factors for PPCS: history of previous TBI (Wojcik, 2014; Morgan et al., 2015), pre-morbid psychiatric disorder (Wojcik, 2014; Morgan et al., 2015; Silverberg et al., 2015; Bramley et al., 2016), pre-morbid headache disorder (Bramley et al., 2016), female gender (Bazarian et al., 2010; Cnossen et al., 2018; Varriano et al., 2018; Underwood et al., 2019), substance misuse

(Jacotte-Simancas et al., 2021) and young age (Babcock et al., 2013). There is also some evidence that older age, i.e., 65+ years (Rothweiler et al., 1998) or 50+ (Varriano et al., 2018), is a risk factor associated with PPCS however there is no consensus (Richey et al., 2020).

Outcomes of mTBI patients presenting within 24 h of injury with a Glasgow Coma Score of 13–15 and whose head trauma history warranted a non-contrast head CT study, were reported in a multicenter study (McMahon et al., 2014). At 3 months following head injury, 33% of patients failed to regain their full functional status and at 12 months after injury up to 25% of patients remained impaired in the workplace or in their daily activities. These trends held regardless of radiographic findings; in some cases, patients with a normal CT experienced significantly worse outcomes compared to patients with an abnormal result. Neuropsychological evaluations using the Brief Symptom Inventory-18 (BSI), Satisfaction with Life Scale and Rivermead Post-Concussion Questionnaire-13 at 6 and 12 months after injury revealed no significant differences at follow up; however, only 53.1% of patients were available for evaluation at the 12-month follow up. These authors concluded the term “mild” is a misnomer for this mTBI population.

### Neuroanatomy of Concussion

Most brain injuries in mTBI result from abrupt acceleration-deceleration and rotational forces on the head (Bigler, 2008). Concussion symptoms are the clinical manifestation of microstructural brain injuries characterized as diffuse axonal injury (DAI) often in association with microhemorrhages (Adams et al., 1989). The secondary effects of DAI are related to populations of neurons facing injured axons or damaged inputs. In contrecoup lesions, for example, the frontotemporal olfactory paralimbic zone is affected with a restricted retrograde impact on ventrolateral thalamus and limbic structures (hippocampus and amygdala) (Koliatsos and Rao, 2020). Bigler emphasized that the lateral surface of the upper brainstem touches the free edge of the tentorium falx cerebelli, while occupying the other side of this part of the falx is the medial surface of the temporal lobe (amygdala and hippocampus). Rotational or stretching forces here, such as in vehicular crashes or high-impact falls, cause the upper brainstem to stretch across the clinoid and lesser wing of the sphenoid impacting the tentorial free edge, the pituitary stalk to stretch disrupting hypothalamic-pituitary connections, and coursing vessels in the area are also affected resulting in perfusion changes to both anterior circulation (to hemispheres) and posterior circulation (to brainstem) (Bigler, 2008). Bigler proposes the biomechanics of concussion result in the vulnerability of the upper brainstem (thalamus), hypothalamic-pituitary axis, medial temporal lobe (hippocampus and amygdala), basal forebrain, long-coursing white matter fibers of the corpus callosum and fornix and concludes these are the brain regions most likely to give rise to postconcussion symptoms. Thus, regardless of presentation as closed head injury, penetrating injury or blast injury, these anatomic relationships appear to be of greater pertinence in the evolution of postconcussion symptoms.

#### Neuropathology

Wallerian degeneration is considered the principal molecular pathology in DAI. Unlike experimental axotomy the biomechanical disruption of axons in TBI leads to varying degrees of pathology, from complete transection and rapid degeneration to no injury at all. Investigators have shown there is a period of time during which injured axons go through a molecular decision process; eventual axonal degeneration occurs anywhere from 4 to 12 h in animal studies and takes even longer in humans. This delay in the commitment of injured axons to Wallerian degeneration is felt to offer a window of opportunity for therapeutic intervention. Because suppression of Wallerian degeneration is achievable by a particular genetic arrangement, it is now thought that axonal survival is not dependent on key support from the cell body. These authors propose that salvageable axons represent the majority of acutely injured axons in DAI and may be restored to normal functionality if key Wallerian degeneration signals are suppressed (Koliatsos and Alexandris, 2019).

### Imaging Studies in Mild Traumatic Brain Injury

Using a magnetic resonance imaging (MRI) technique termed symmetric normalization for multivariate neuroanatomy (SyNMN) investigators provided evidence that TBI lesions significantly compromise paralimbic structures (dorsomedial thalamus and hippocampus) (Avants et al., 2008). Multimodal studies using magnetic resonance imaging have recently improved the more widespread and specific detection of DAI injuries with notable consistent mention of these same paralimbic structures (Lunkova et al., 2021). Posttraumatic (non-concussive) stress disorder (PTSD) is a psychiatric condition sharing similar symptoms of postconcussion syndrome and when both disorders co-exist in post-combat veterans the condition is conceptualized as “consequence of war syndrome” (CWS) (Dieter and Engel, 2019). Symptoms of PTSD are thought to reflect a dysfunction of the task-free large-scale networks of the brain (“resting state networks”), namely the salience, central executive and default mode networks (DMN) (Fenster et al., 2018). In a study of 102 male veterans with PTSD using structural and diffusion MRI techniques, severity of PTSD symptoms correlated with abnormalities in the right amygdala-hippocampus complex, right cingulate cortex and left medial orbitofrontal cortex. In a PTSD plus mTBI cohort, symptoms severity correlated with bilateral involvement of these same structures (Sydnor et al., 2020). The authors theorize that PTSD results from non-concussive, stress-induced loss of neuronal connectivity in these paralimbic regions.

In a study of patients diagnosed with uncomplicated mTBI (see Table 1) presenting within 7 days of injury, fMRI performed at entry and at 6 months (*n* = 25) demonstrated reduced functional connectivity at presentation in multiple networks: anterior default mode network (frontal pole), central executive network (postcentral gyrus), somatomotor network (precentral gyrus and cingulate) and auditory network (insular cortex and postcentral gyrus). Consistently, network connectivity was negatively correlated with severity of postconcussion symptoms (D’Souza et al., 2020). Reduced network connectivity in these specific resting state networks was correlated with severity of persistent postconcussion symptoms (PPCS), and an increase in network connectivity at 6 months correlated with symptom improvement. These authors concluded that mTBI is associated with functional brain network abnormalities that evolve over time and may contribute to cognitive deficits and PCS severity. Using a co-registration technique based on functional alignment of structurally based DMN maps (such as those depicted in the aforementioned study) other investigators have built a more comprehensive DMN model to include the contribution of subcortical structures. They demonstrated both limbic thalamus and basal forebrain as nodes with high degree and high centrality in the DMN, noting that injury to these structures would lead to a drastic decrease of functional connectivity in the whole DMN (Alves et al., 2019).

### Treatment

There are no specific treatments for PCS/PPCS or cure for the underlying neuropathology. Current medical management consists of measures aimed primarily at symptom relief. “Treatment as usual” in this study comprises pharmacologic interventions, rehabilitation services and referrals for cognitive behavioral therapy.

#### Neuromodulation for Mild Traumatic Brain Injury

Neuromodulation is the term used for training brain behavior by encouraging adaptive neuroplasticity. Several studies have explored the benefits provided by varying neurofeedback (NF) modalities in post-concussion syndrome. Alpha-theta NF was found to benefit symptom reduction, perceived stress and serum cortisol levels in 60 patients with a history of MVA-related TBI (Bennett et al., 2018) as well as one 30-year old male patient with history of mild TBI in the areas of verbal and visual learning and memory (Reddy et al., 2009). Low energy neurofeedback system (LENS) training was associated with spontaneous reversal of chronic anosmia secondary to acceleration-deceleration TBI in two patients (Hammond, 2008). A one-subject case study showed clinical benefits obtained from traditional QEEG-based NF following two consecutive concussions, treated with 20 and 40 sessions of NF respectively (Linden, 2015). Additionally, significant improvements in cognitive scores and concussion symptoms as well as neurophysiological and functional connectivity changes were found in a study of two patients presenting with moderate TBI, extensive symptoms and poor cognitive performance who underwent 20 sessions of alpha-theta training over the course of 4 weeks (Munivenkatappa et al., 2014). In a 30-month follow up study, 12 of 15 Vietnam veterans with combat-related PTSD did not experience a relapse after receiving alpha-theta brainwave NF compared to 14 veterans receiving treatment as usual who all experienced a relapse (Peniston and Kulkosky, 1991). A report on different neuromodulation techniques for mTBI described positive findings in 13 of 14 papers reviewed, concluding that neuromodulation warrants further investigation (Buhagiar et al., 2020).

The choice for infra-low frequency (<0.01 Hz) neuromodulation is to more directly engage with large-scale brain networks that lie below the networks of cognition or lateralization; many of these networks have been characterized in fMRI studies, most notably the DMN. Infralow frequency (ILF) brain training, the method we use in this study, has been shown to enhance the quality of life of veterans with mTBI by alleviating their chronic postconcussion symptoms, with significant clinical gains found in self-reported chronic headaches, insomnia and attentional difficulties (Carlson and Webster Ross, 2021).

#### Theory of Mechanism

ILF neurotherapy directly engages with the infraslow oscillatory networks in the brain encompassing the so-called “slow control system,” as described by Aladjalova (1957). Aladjalova was able to evoke this infraslow activity after 20 min of stimulating the posterior hypothalamus in the rabbit brain. The hypothalamus itself is responsible for wholescale brain homeostasis and it works in concert with the limbic structures to regulate emotions, mood and behavior as well as attention, drive, memory and learning. When we strengthen these regulatory networks in the dysregulated brain, such as may follow concussion injury, we are bound to improve the major post-concussion symptoms (headache, memory impairment, brain fog, sleep disturbances, fatigue, stress) that actually reflect a disturbance of hypothalamic-limbic regulation.

The DMN is a slow oscillatory network, among other resting state task-negative networks, and the usual “basic” sites in ILF neurotherapy are positioned within the regions of the DMN (Othmer and Othmer, 2020). Disruptions of the default mode network have been studied in multiple conditions including depression, ADHD, epilepsy and mild cognitive impairment (Broyd et al., 2009). Researchers theorize that modulating neuronal activity at the network level, such as the DMN, is more effective than targeting one area of the brain with such training (and more effective than the use of medications) (Sitaram et al., 2017). While we agree with this generalization our experience and current study findings suggest that in the realm of concussion, training at the level of the DMN may cause additional irritation, as has been our experience when attempting to train at basic scalp sites like T4-P4. This is not surprising, as fMRI investigations have consistently demonstrated that even mild TBI significantly impairs functional connectivity of the anterior DMN and other resting state networks (RSN) (D’Souza et al., 2020). Specifically in postconcussion cases, our objective with ILF neurotherapy is to first address long-distance axonal connections beyond the regions of the identifiable networks like the DMN while remaining inclusive of their domains. Notably, alpha-theta training at O1+O2 (not a basic scalp site used in ILF training) has been successfully performed by many neurofeedback clinicians on patients with mTBI (Gupta et al., 2020).

Our study sought to investigate for efficacy of ILF training in PPCS and to provide comparative discernment to treatment as usual paradigms, a method lacking in most postconcussion neurofeedback research. A concussion protocol developed in our lab, following careful observation and successive responses to ILF training in one of our patients with severe PPCS (Legarda, 2020) and thereafter adopted generally for patients with PPCS, was assessed for any additional efficacy.

## Method

### Study Design

This retrospective study was conducted with a lookback of 2.5 years. Subjects carrying the medical diagnosis of postconcussion syndrome and/or history of concussion were selected. Two treatment modalities were received by the study sample: treatment as usual (TAU) or treatment as usual with integrative ILF neurotherapy (TAU+ILF). We investigated pre- vs. post-treatment symptom profiles within each treatment modality. Treatments were assessed for individual as well as comparative efficacy in symptom improvement and/or resolution. All co-investigators were trained in conducting human research and approval from our local institutional review board was obtained to perform the study.

### Subject Selection

All subjects were seen at the Montage Health neurology group consisting of five independently practicing neurologists. An electronic medical record database search was implemented seeking patients with the following criteria: (1) ICD-10 diagnosis codes of F07.81 (“postconcussion syndrome,” “post-traumatic brain syndrome, non-psychotic,” “chronic traumatic encephalopathy”) or Z87.280 (“history of concussion” or “history of multiple concussions”), (2) referral to neurology, and (3) seen within the time frame of 08/01/2018 to 01/31/2021. This timeframe was selected to encompass two years plus the six months lost to the slowdown of clinic activity during the COVID-19 pandemic. Through retrospective chart review, study subjects were divided into two groups: TAU (*n* = 50, *n* = 27 females, mean age 47.5 ± 20.7 years) and TAU+ILF (*n* = 33, *n* = 18 females, mean age 51.2 ± 22.0 years).

Inclusion criteria for the TAU group were: (1) diagnosis of F07.81 or Z87.280, (2) received treatment as usual (i.e., did not receive ILF neurotherapy), and (3) first office visit on or between 08/01/18 and 01/31/21. Patients were excluded from the TAU group if the patient was lost to follow up (i.e., presented for initial office visit, did not show for follow up, and was called and unreachable within 6 months of last visit). There were two patients in the TAU group whose follow up was conducted via telephone after expressing informed consent.

**FIGURE 1:**
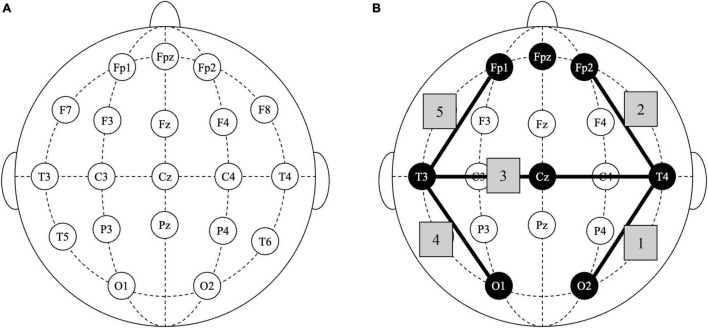
Infralow frequency neurofeedback: concussion protocol. (A) Standard 10–20 EEG nomenclature diagram of scalp sites for electrode placement. (B) Diagram of selected scalp placements in concussion protocol; shaded nodes indicate all electrode placements used; shaded edges (1–5) indicate sequential positioning of consecutive bipolar lead electrodes: T4-O2, T4-FP2, T4-T3, T3-O1, and T3-FP1.

Inclusion criteria for TAU+ILF were: (1) coded diagnosis of F07.81 or Z87.280, (2) referred for neurofeedback, (3) first neurofeedback session on or between 08/01/18 and 01/31/21, and (4) at least 10 total sessions of ILF neurofeedback training. Subgroups of TAU+ILF were identified as follows: patients who were started on concussion protocol (CP, see Figure 1B) and received at least 10 sessions of CP (TAU+ILF+CP) and those who either were not started on CP or received less than 10 sessions of CP (TAU+ILF-CP).

Patients were not excluded based on age, gender, time to treatment, number of concussions or concussion severity for the purposes of obtaining a complete picture of the generalized efficacy of ILF brain training. This is with one exception: one patient sustained a new concussion preceding several consecutive follow-up visits with neurology and was therefore excluded due to consistent interruption of symptom tracking. Additionally, one subject was excluded due to treatment non-compliance of both treatment as usual and ILF.

### Method of Infra-Low Frequency Brain Training

In our clinic the patient sits comfortably in a chair, with leg rest, in a dark and quiet room, and chooses a DVD movie of their choice to view on a high-resolution 42” monitor. The patient’s head is measured according to 10–20 EEG nomenclature (see Figure 1A). Prior to training, selected scalp sites are prepared with a mild abrasive gel (Nuprep) to clean the skin surface, before applying silver-coated electrodes using conductive electrode paste (Ten20) to ensure adherence to scalp locations. Two scalp electrodes are placed at Fpz, used as ground reference and at Cz, used as common reference. Brain activity is recorded in bipolar differential technique by a NeuroAmp II neuroamplifier with firmware (digitizing the raw signal) and transmitted to an upgraded computer system compatible with Cygnet software.^1^ (An example of bipolar recording method: (T4-Cz)°-°(O2-Cz) is used when recording from scalp sites T4-O2.) Cygnet uses a band pass filter centered around the slow cortical potential (SCP) (Aladjalova, 1957) and target frequencies extend below 0.1 mHz. The two recording electrodes may be sequentially moved to successive scalp locations according to the brain training protocol prescribed.

The action of the neurofeedback loop is covert in this set-up; the signal recorded from the brain and presented back to the patient via the monitor is embedded in the visual imagery of interest to the patient who is not actively aware of the specific training frequency (unlike traditional forms of neurofeedback). Continuity and strengthening of the feedback loop are contingent upon the patient’s brain detecting its agency in the set-up. In each successive training session, the frequency selected for reinforcement is lowered. The goal of decreasing the reinforcement frequency is to train wider and more intrinsic connectivity in the brain; the slower the reinforcement frequency the more likely the client engages with their earliest networks and adaptive neuroplasticity dynamics (Legarda, 2020). An optimal reinforcement frequency is realized in some patients wherein lowering the frequency training further is not tolerated.

Specific to this study, the two scalp electrodes selected for “bipolar training” were placed at different sites every 10 min in a stepwise progression in accordance with the patient’s prescribed brain training protocol, for a total length of 50 min per session. The concussion protocol, a focus in this study, is depicted numerically in Figure 1B.

### Measures

#### Symptom Quantification

Symptoms and symptom severity were quantified before, during and after treatment as documented by a neurologist at each initial and subsequent visit. Symptoms tracked were selected according to the Rivermead Post-concussion Questionnaire (RPQ) (King et al., 1995), with the addition of two post-concussion symptoms which we felt were either relevant or of high frequency among our patient population: nervousness/anxiety (*n* = 29) and seizures (*n* = 4). The complete list of PPCS symptoms considered was as follows: headache, dizziness, nausea/vomiting, phonophobia, photophobia, sleep changes, fatigue, irritability, sadness/depression, easy frustration, poor memory, difficulty concentrating, brain fog/poor cognition, blurred vision, double vision, restlessness, nervousness/anxiety, and seizures.

#### Cognitive Assessment

The Montreal Cognitive Assessment (MoCA) (Nasreddine et al., 2005) and/or Mini Mental Status Exam (MMSE) (Folstein et al., 1975) questionnaires, while inconsistently administered, were recorded and analyzed in depth for possible relevance.

### Statistical Analyses

Median and interquartile range were reported for non-normally distributed data, e.g., number of symptoms, while mean and standard deviation were used for normally distributed data, e.g., age. The Wilcoxon Signed Rank test was used to compare number of symptoms pre- and post-intervention within treatment groups. The Mann Whitney U test was used to compare treatment efficacy between groups: TAU vs. TAU+ILF, TAU vs. TAU+ILF+CP, TAU vs. TAU+ILF-CP, and TAU+ILF+CP vs. TAU+ILF-CP. The Mann Whitney *U* test was also used to compare efficacy among type and quantity of ILF sessions.

A binary logistic regression assessed possible impacts of independent variables previously reported to have significance in the course of post-concussion syndrome (TBI severity (McMahon et al., 2014), history of previous TBI (Wojcik, 2014; Morgan et al., 2015), pre-morbid psychiatric disorder (Wojcik, 2014; Morgan et al., 2015; Silverberg et al., 2015; Bramley et al., 2016), pre-morbid headache disorder (Bramley et al., 2016), gender (Bazarian et al., 2010; Cnossen et al., 2018; Varriano et al., 2018; Underwood et al., 2019), age (Rothweiler et al., 1998; Babcock et al., 2013; Varriano et al., 2018) and substance misuse (Jacotte-Simancas et al., 2021), in comparing outcomes within and between TAU and TAU+ILF treatment groups. All independent variables were assessed for collinearity before inclusion in the regression.

Chi square analyses using Fisher’s Exact Test were also performed for a second line of statistical investigation of independent variables. Fisher’s Exact Test was used due to small sample size.

From patient histories we determined severity of their head injury using Table 2 criteria below.

**TABLE 2:** Measures of TBI severity (Brasure et al., 2012).

Criteria	Mild	Moderate	Severe
Structural imaging	Normal	Normal or abnormal	Normal or abnormal
Loss of consciousness	<30 min	30 min to 24 h	>24 h
Alteration of consciousness/mental state	A moment to 24 h	>24 h	>24 h
Post-traumatic amnesia	0–1 day	>1 and <7 days	>7 days
Glasgow Coma Scale (best available score in 24 h)	13–15	9–12	3–8

## Results

**FIGURE 2:**
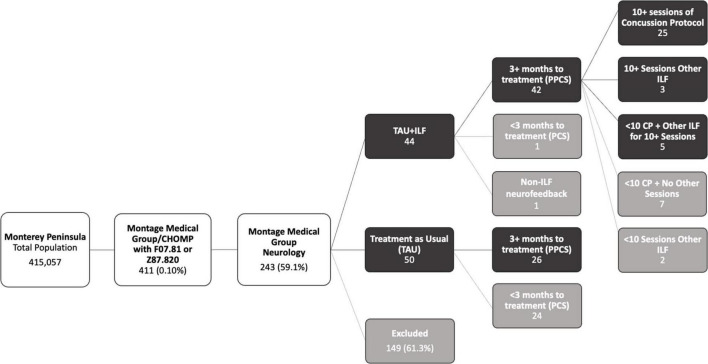
Flowchart of population breakdown to groups and subgroups.

A total of 411 patients carried the diagnoses of F07.81 (post-concussion syndrome) or Z87.820 (history of concussion) between the dates of 08/01/2018 and 01/31/2021, representing a small prevalence of 0.10% in our Monterey Peninsula community (see Figure 2). Of these, 243 patients (59.1%) were referred to a neurologist for further evaluation. A total of 84 patients met inclusion criteria for this study, 33 patients in the infralow feedback (TAU+ILF) group and 50 cases in the treatment as usual (TAU) group, while 159 were necessarily excluded (see listed reasons for exclusion in Supplementary Table 1).

All but one patient who received ILF qualified for PPCS. Among the 50 TAU patients who passed inclusion criteria, 26 qualified for PPCS while the rest did not. Therefore, for relevance of comparison, only TAU+ILF and TAU patients qualifying for PPCS diagnosis were included in further analyses. Included subjects were further characterized into the following subgroups within the TAU+ILF group: ILF using 10 sessions of concussion protocol (TAU+ILF+CP) and all other 10 sessions of ILF cases (TAU+ILF-CP). Demographic information of these groups is summarized in Table 3.

**TABLE 3:** Summary of included PPCS patients by group.

Treatment group	*n*	*n* female (%)	Age (mean ± SD)
TAU	26	12 (46.2%)	52.0 ± 21.2
TAU+ILF	33	18 (54.5%)	51.2 ± 22.0
TAU+ILF+CP	25	13 (52.0%)	53.0 ± 22.8
TAU+ILF-CP	8	5 (62.5%)	45.6 ± 19.8

*All patients met inclusion criteria for this study as well as diagnostic criteria for persistent post-concussion symptoms: symptoms lasting greater than 3 months post-injury before initiation of treatment.*

### Symptom Quantification

**FIGURE 3:**
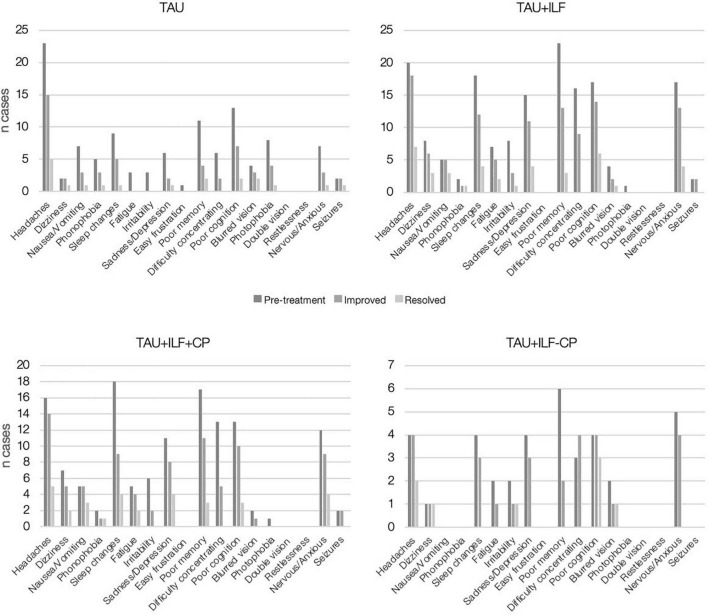
Persistent post-concussion symptom progression by symptom and treatment group.

Symptoms and symptom severity were quantified before, during and after treatment as documented by neurology physicians. Figure 3 displays total number of cases per symptom and overall symptom progression within each treatment group. The most prevalent presenting PPCS symptoms were headache (*n* = 40, 67.8% of patients), poor memory or forgetfulness (*n* = 34, 57.6% of patients), and brain fog/poor cognition (*n* = 30, 50.8% of patients). The TAU treatment group saw the largest percent resolution among the following symptoms: dizziness (50.0%), blurred vision (50.0%), and seizures (50.0%); TAU+ILF: nausea/vomiting (60.0%), phonophobia (50.0%), and dizziness (37.5%); TAU+ILF+CP: nausea/vomiting (60.0%), phonophobia (50.0%), and fatigue (40.0%); TAU+ILF-CP: dizziness (100.0%), poor cognition (75.0%), and headaches (50.0%), irritability (50.0%), and blurred vision (50.0%).

Table 4 summarizes time to treatment, time to first symptom improvement and time to first symptom resolution among our treatment groups. Most treatment groups demonstrated comparable median times to treatment (1.7–1.8 years) with the exception of TAU+ILF-CP (3.8 years). The TAU+ILF group exhibited a faster median time to first symptom improvement (TAU+ILF: 2.5 months, TAU+ILF+CP: 2.6 months, and TAU+ILF-CP: 2.6 months) compared to TAU alone (3.0 months). Median time to first symptom resolution was comparable between TAU (4.6 months) and TAU+ILF+CP (4.8 months) but extended for TAU+ILF-CP (1.1 years).

**TABLE 4:** Duration of symptoms until treatment, first recorded improvement or resolution among persistent post-concussion patients.

Treatment	Median time to treatment	n Improved (% of group)	Median time to 1st improvement	*n* Resolved (% of group)	Median time to 1st resolution
TAU (*n* = 26)	1.8 years	22 (84.6%)	3.0 months	10 (38.5%)	4.6 months
TAU+ILF (*n* = 33)	1.8 years	32 (97.0%)	2.5 months	17 (51.5%)	5.4 months
TAU+ILF+CP (*n* = 25)	1.7 years	25 (100.0%)	2.6 months	13 (52.0%)	4.8 months
TAU+ILF-CP (*n* = 8)	3.8 years	7 (87.5%)	2.4 months	4 (50.0%)	1.1 years

*Values reflect patients who qualified for “improved” or “resolved”—calculations do not include patients who did not experience symptom improvement or resolution.*

### Cognitive Assessments

Montreal Cognitive Assessment (MoCA) questionnaires were completed pre- and post-treatment by five individuals in the TAU group and two individuals in the TAU+ILF-CP group, 0 of whom also completed a pre- and post-treatment Mini Mental Status Examination (MMSE). In the TAU+ILF+CP group, 12 individuals completed pre- and post-treatment measures for MoCA, 9 of whom also completed a pre- and post-treatment MMSE. Supplementary Tables 2, 3 summarize the results of these measurements. No significant changes in MoCA or MMSE score were noted in any treatment group as determined via the Wilcoxon Rank Sum test.

A binary logistic regression was performed to assess for variables which may impact MoCA improvement such as age, gender, time to treatment, history of multiple concussions, anxiety, depression, PTSD, substance misuse and TBI severity, however none tested were found to have significant impact on MoCA outcomes. A chi square test was also used to assess for impacts of the same independent variables on MOCA results. Under chi square it was found that patients with a documented history of or current substance dependence were less likely to improve their scores than expected (Supplementary Table 4); this result met an asymptotic significance threshold of *p* < 0.05 but did not meet this significance threshold under Fisher’s Exact Test.

Overall, 13 of 19 patients receiving pre- and post-treatment MoCA exhibited improved scores (68.4%). Of these patients, 9 improved with TAU+ILF+CP treatment (75.0%), and 3 did not (25.0%); 3 improved with TAU treatment (60.0%), and 2 did not (40.0%) and 1 improved with TAU+ILF-CP treatment (50.0%), while 1 did not (50.0%).

### Individual Group Analyses

Table 5 below summarizes the efficacy of individual treatments by comparing symptom quantities before and after treatment. Treatment as usual (TAU) showed statistical significance as an efficacious standalone treatment in both the improvement (*p* < 0.001) and resolution (*p* = 0.007) of persistent post-concussion symptoms. Among TAU+ILF subgroups, statistical significance is observed in the TAU+ILF+CP subgroup in both improvement (*p* < 0.001) and resolution (*p* = 0.001) of symptoms, while the TAU+ILF-CP subgroup demonstrated significance only in improvement of symptoms (*p* = 0.017).

**TABLE 5:** Pre- vs. post-treatment symptom comparisons within individual treatment types.

Treatment	n	Median (Q1–Q3)	Symptom effect	Median (Q1–Q3)	Significance (*p*)
		pre-treatment symptoms		affected symptoms	
TAU	26	4 (3.25–5)	Improved	1 (1–3.75)	<0.001
			Resolved	0 (0–1)	0.007
TAU+ILF	33	5 (4–7)	Improved	3 (2–5)	<0.001
			Resolved	1 (0–2)	<0.001
TAU+ILF+CP	25	5 (4–7)	Improved	3 (2–5)	<0.001
			Resolved	1 (0–2)	0.001
TAU+ILF-CP	8	4 (3–6.25)	Improved	3 (2.5–4.5)	0.017
			Resolved	0.5 (0–2)	0.066

*A Wilcoxon Rank-Sum test was used to assess pre- vs. post-treatment symptom quantity within each treatment group: TAU, TAU+ILF, and TAU+ILF subgroups (TAU+ILF+CP, TAU+ILF-CP). The test assessed for either symptom improvement (“Improved”) or symptom resolution (“Resolved”) following treatment. Statistically significant values (p < 0.05) indicate positive association of the treatment with symptom improvement or resolution in each group; these are highlighted in red.*

### Comparative Group Analyses

Comparative analyses were conducted to assess differences in symptom improvement or resolution between TAU+ILF, its subgroups, and TAU. Tables 6, 7 highlight findings from tests comparing quantity of symptoms improved or percent of symptoms improved, respectively, with significant results (*p* < 0.05) indicating one treatment was associated with greater net or percent symptom improvement when compared to the other treatment. Supplementary Tables 5, 6 display results from tests comparing net and percent of symptoms resolved, respectively. To indicate the direction of significance, mean ranks are reported, with a greater mean rank indicating greater overall symptoms improved or resolved and therefore resulting in a trend of TAU+ILF > TAU or TAU+ILF < TAU (see “Trend” column).

**TABLE 6:** Comparative analysis of TAU vs. TAU+ILF and subgroups in the improvement of PPCS symptoms.

Groups	n	Median (Q1-Q3)	Mean Rank	Sum of Ranks	U	*p*	Trend
		Δ Symptoms					
TAU	26	1 (1–3.75)	22.98	597.50	246.50	0.005	ILF > TAU
TAU+ILF	33	3 (2–5)	35.53	1172.50			

TAU	26	1 (1–3.75)	20.29	527.50	176.50	0.004	ILF > TAU
TAU+ILF+CP	25	3 (2–5)	31.94	798.50			

TAU	26	1 (1–3.75)	16.19	421.00	70.00	0.158	ILF > TAU
TAU+ILF-CP	8	3 (2.5–4.5)	21.75	174.00			

TAU+ILF+CP	25	3 (2–5)	17.12	428.00	97.00	0.898	+CP > -CP
TAU+ILF-CP	8	3 (2.5–4.5)	16.63	133.00			

*A Mann-Whitney U test assessed differences in improved symptom quantities between treatment groups. The following comparisons were made: TAU vs. TAU+ILF, TAU vs. TAU+ILF+CP, TAU vs. TAU+ILF-CP, and TAU+ILF+CP vs. TAU+ILF-CP. Statistically significant values (p < 0.05) indicate tendency for greater number of symptoms improved or resolved in one of the compared groups; these are highlighted in red. The “Trend” column highlights the direction of greater value in the comparison, e.g., whether the overall number of improved or resolved symptoms was greater for the TAU+ILF (sub)group or TAU. The TAU+ILF group and its subgroups are abbreviated to “ILF” in the Trend column.*

**TABLE 7:** Comparative analysis of TAU vs. TAU+ILF and subgroups in the percent improvement of PPCS symptoms.

Groups	n	Median (Q1-Q3)	Mean Rank	Sum of Ranks	U	*p*	Trend
		% Δ Symptoms					
TAU	26	41.4% (25.0–95.0%)	24.62	640.00	289.00	0.029	ILF > TAU
TAU+ILF	33	83.3% (50.0–100%)	34.24	1130.00			

TAU	26	41.4% (25.0–95.0%)	21.56	560.50	209.50	0.026	ILF > TAU
TAU+ILF+CP	25	83.3% (50.0–100%)	30.62	765.50			

TAU	26	41.4% (25.0–95.0%)	16.56	430.50	79.50	0.312	ILF > TAU
TAU+ILF-CP	8	75.0% (51.2–100%)	20.56	164.50			

TAU+ILF+CP	25	83.3% (50.0–100%)	17.26	431.50	93.50	0.778	+CP > -CP
TAU+ILF-CP	8	75.0% (51.2–100%)	16.19	129.50			

*A Mann-Whitney U test assessed differences in percentage of symptoms improved between treatment groups. The following comparisons were made: TAU vs. TAU+ILF, TAU vs. TAU+ILF+CP, TAU vs. TAU+ILF-CP, and TAU+ILF+CP vs. TAU+ILF-CP. Statistically significant values (p < 0.05) indicate tendency for greater percent symptom improvement or resolution in one of the compared groups; these are highlighted in red. The “Trend” column highlights the direction of greater value in the comparison, e.g., whether the overall percentage of improved or resolved symptoms was greater for TAU or TAU+ILF. The TAU+ILF group and its subgroups are abbreviated to “ILF” in the Trend column.*

After calculating the number of symptoms pre- and post-treatment, significant distinctions were found when comparing the TAU+ILF group vs. TAU (*p* = 0.005) and the TAU+ILF+CP subgroup vs. TAU (*p* = 0.004) in net improvement of symptoms (Table 6), but not in net symptom resolution (Supplementary Table 5). Significant results were also found comparing TAU+ILF vs. TAU (*p* = 0.029) and TAU+ILF+CP vs. TAU (*p* = 0.026) in percent symptom improvement (Table 7) but not in percent symptom resolution (Supplementary Table 6). There was a trend in greater net resolution of symptoms in both TAU+ILF vs. TAU and TAU+ILF+CP vs. TAU (Supplementary Table 5), but a trend of greater percent resolution of symptoms only in TAU+ILF+CP vs. TAU (Supplementary Table 6). Trends of greater net and percent symptom improvement and resolution were seen when comparing TAU+ILF-CP over TAU; however, these findings did not meet our threshold for significance. Finally, trends toward greater net and percent symptom improvement among TAU+ILF+CP subjects were noted over those in TAU+ILF-CP, however these trends were not found to be significant.

### Neurofeedback Session Analysis

Discrepancies in symptom remediation in relationship to number and type of neurofeedback sessions were analyzed via Mann Whitney U Test. We did not find significant differences in improvement or resolution between patients receiving at least 10 sessions of concussion protocol over patients receiving less than 10 sessions of said protocol before switching to another protocol. Therefore, we assessed subjects receiving at least 20, 30, or 40 sessions of concussion protocol compared to subjects receiving < 20, < 30, or < 40 sessions of concussion protocol and to subjects receiving 20+, 30+, or 40+ sessions of any non-concussion protocols. Significance in net symptom improvement (Table 8) was noted at the level of 40+ sessions of concussion protocol over < 40 sessions (*p* = 0.011), as well as 30+ and 40+ sessions of concussion protocol over 30+ (*p* = 0.053) and 40+ sessions (*p* = 0.024) of other protocols. Receiving 40+ sessions of concussion protocol was also associated with significant percent symptom improvement over ILF patients receiving 40+ sessions of other protocols (p = 0.027, Table 9). Otherwise, all other trends were non-significant in our findings; comparison results of net and percent symptom resolution are located in Supplementary Tables 7, 8. To summarize, patients training at least 40 sessions of concussion protocol (*n* = 3) showed greater net (*p* = 0.024) and percent improvement (*p* = 0.027) of symptoms than patients training for at least 40 sessions with other protocols (*n* = 6); greater significance of symptom improvement in concussion protocol over other protocols was seen in those who trained 40 sessions.

**TABLE 8:** Comparative analysis of number and type of neurofeedback sessions on symptom improvement status.

Protocol comparisons	n	Median (Q1-Q3)	Mean Rank	Sum of Ranks	U	*p*	Trend
		Δ symptoms					
1–9 CP	5	3 (3–4)	15.00	75.00	60.00	0.888	10+ > <10
10+ CP	25	3 (2–5)	15.60	390.00			

1–19 CP	20	3 (2–4.25)	14.50	290.00	80.00	0.371	20+ > <20
20+ CP	10	4 (2.25–5)	17.50	175.00			

1–29 CP	26	3 (2–4)	14.44	375.50	24.50	0.088	30+ > <30
30+ CP	4	5.5 (4.25–6)	22.38	89.50			

1–39 CP	27	3 (2–4)	14.17	382.50	4.50	0.011	40+ > <40
40+ CP	3	6 (5.5–6)	27.50	82.50			

20+ Any	11	3 (2–4.5)	10.50	126.00	48.00	0.421	CP > Any
20+ CP	10	4 (2.25–5)	12.70	127.00			

30+ Any	11	2 (2–3.5)	6.68	73.50	7.50	0.053	CP > Any
30+ CP	4	5.5 (4.25–6)	11.63	46.50			

40+ Any	6	2 (2–2.75)	3.58	21.50	0.50	0.024	CP > Any
40+ CP	3	6 (5.5–6)	7.83	23.50			

*The number and type of neurofeedback sessions were analyzed for discrepancies in net symptom improvement. Analysis was conducted via Mann Whitney U test.*

**TABLE 9:** Comparative analysis of number and type of neurofeedback sessions on percent symptom improvement.

Protocol comparisons	n	Median (Q1-Q3) % Δ Symptoms	Mean Rank	Sum of Ranks	U	*p*	Trend
1–9 CP	5	75.0% (57.1–100%)	15.00	75.00	60.00	0.885	10+ > <10
10+ CP	25	83.3% (50.0–100%)	15.60	390.00			

1–19 CP	20	79.2% (50.0–100%)	15.68	313.50	96.50	0.873	< 20 >20+
20+ CP	10	84.5% (44.6–100%)	15.15	151.50			

1–29 CP	26	79.2% (50.0–100%)	15.50	403.00	52.00	1.000	30+ = <30
30+ CP	4	84.5% (69.6–89.3%)	15.50	62.00			

1–39 CP	27	75.0% (50.0–100%)	15.06	406.50	28.50	0.390	40+ > <40
40+ CP	3	85.7% (84.5–92.9%)	19.50	58.50			

20+ Any	11	75.0% (50.0–91.7%)	10.64	117.00	51.00	0.774	CP > Any
20+ CP	10	84.5% (44.6–100%)	11.40	114.00			

30+ Any	11	50.0% (41.2–75.0%)	7.14	78.50	12.50	0.212	CP > Any
30+ CP	4	84.5% (69.6–89.3%)	10.38	41.50			

40+ Any	6	39.3% (28.6–68.8%)	3.58	21.50	0.50	0.027	CP > Any
40+ CP	3	85.7% (84.5–92.9%)	7.83	23.50			

*The number and type of neurofeedback sessions were analyzed for discrepancies in percent symptom improvement. Analysis was conducted via Mann Whitney U test.*

### Regression Analysis

The effects of possible confounding factors on symptom improvement and resolution were analyzed via binary logistic regression. All factors were found to be non-significant in their effects on symptom improvement, so these results have been supplied in Supplementary Table 9. The table below discusses regression results over symptom resolution only, including all PPCS patients (*n* = 26 TAU, *n* = 25 TAU+ILF+CP, *n* = 8 TAU+ILF-CP). Supplementary Table 10 limits the regression to PPCS patients receiving TAU only. The TAU+ILF only regression was unable to be computed because a perfect fit was detected, and the solution was determined to be non-unique.

Which treatment subjects received was non-significant with all other factors considered in the overall regression (Table 10). Patients with a concurrent diagnosis of PTSD were significantly more likely to experience symptom resolution than patients without this diagnosis in the overall PPCS regression (*p* = 0.015, Table 10). Patients without a concurrent diagnosis of anxiety were associated with significantly increased likelihood of symptom resolution than patients with anxiety in the overall regression (*p* = 0.005, Table 10). Subjects aged <25 years old were more likely to resolve their symptoms than patients aged 65+ years (*p* = 0.007). Finally, patients with mild TBI were significantly more likely to experience symptom resolution than patients with severe TBI in the overall PPCS regression (*p* = 0.015). No independent variables resulted in any level of significance in the TAU only logistic regression (Supplementary Table 10).

**TABLE 10:** Binary logistic regression of possible confounding factors on persistent post-concussion symptom resolution (*n* = 59).

				95% C.I. for OR	
				
		Sig.	Odds ratio (OR)	Lower	Upper
Independent variable	Treatment (ref. TAU+ILF-CP)	0.571			
	TAU	0.588	0.429	0.020	9.194
	TAU+ILF+CP	0.968	1.075	0.031	36.933
	PTSD (ref. yes)	0.015	0.005	0.000	0.358
	Depression (ref. yes)	0.611	1.808	0.185	17.716
	Anxiety (ref. yes)	0.003	5577.415	19.734	1.576 × 10^6^
	Age range (ref. 65+ y.o.)	0.013			
	<25 y.o.	0.007	230.599	4.294	1.238 × 10^4^
	25–65 y.o.	0.781	0.739	0.088	6.227
	Gender (ref. female)	0.018	0.070	0.008	0.640
	History of multiple concussions (ref. yes)	0.432	0.420	0.048	3.648
	TBI severity (ref. Severe)	0.046			
	Mild	0.015	43.944	2.091	923.372
	Moderate	0.078	31.343	0.684	1436.834
	History of/present substance misuse (ref. yes)	0.439	0.330	0.020	5.486
	Constant	0.136	0.030		
Model statistics	Percentage of accuracy of classification	86.4%			
	Model significance	<0.001			
	Nagelkerke R square	0.632			
	Hosmer and Lemeshow test sig.	0.015			

*A binary logistic regression assessed for variable effects on symptom resolution among persistent post-concussion patients (n = 59). The dependent variable was set to occurrence of symptom resolution (0 = no, 1 = yes).*

### Chi Square Analyses

The effects of possible confounding factors on symptom improvement and resolution were analyzed via chi square analysis. Fisher’s Exact Test was reported and used for assessing significance when applicable. Significant values are reported below; all other results are reported in Supplementary Tables 11–19. Significant differences in probability of symptom improvement or resolution were not seen between TAU, TAU+ILF+CP, or TAU+ILF-CP. Subjects 65+ years of age were less likely to exhibit symptom improvement than expected than those aged 25–65 and <25 years, regardless of treatment type (*p* = 0.029, Table 11) and within the TAU+ILF-CP group (*p* = 0.018, Table 11). However, this significance did not persist when limiting the patient pool to TAU or TAU+ILF+CP. Diagnosis of pre-injury anxiety was associated with decreased likelihood of symptom resolution regardless of treatment type (*p* = 0.007, Table 12). Finally, a documented substance dependence was associated with fewer instances of symptom improvement than expected in the TAU+ILF patient group, which was found to be significant asymptotically (*p* < 0.001) but did not meet the significance threshold under Fisher’s Exact Test (*p* = 0.061, Table 11). One hundred percent of TAU+ILF+CP patients experienced at least one symptom improvement; therefore, chi square analyses could not be computed within these parameters. No independent variables were found to have significant impacts on symptom resolution within the TAU+ILF group or its TAU+ILF+CP and TAU+ILF-CP subgroups (Supplementary Tables 17–19, respectively).

**TABLE 11:** Chi square analysis of independent variable effects on symptom improvement among various subgroups of PPCS patients.

		Symptom improvement?				
		
Treatment group		No	Yes	χ^2^ (df, N)	Asymptotic sig. (2-sided)	Fisher’s Exact Test (2-sided)
		Count (Expected)	Count (Expected)			
All PPCS	Age range			7.062 (2, 59)	0.029	–
	<25 y.o.	0 (0.8)	9 (8.2)			
	25–65 y.o.	1 (2.8)	32 (30.2)			
	65+ y.o.	4 (1.4)	13 (15.6)			
TAU	History of multiple concussions			4.342 (1, 26)	0.037	0.072
	No	1 (2.8)	17 (15.2)			
	Yes	3 (1.2)	5 (6.8)			
TAU+ILF	Pre-injury anxiety			5.775 (1, 33)	0.016	0.152
	No	0 (0.8)	28 (27.2)			
	Yes	1 (0.2)	4 (4.8)			
	History of or current substance dependence			15.984 (1, 33)	<0.001	0.061
	No	0 (0.9)	31 (30.1)			
	Yes	1 (0.1)	1 (1.9)			
TAU+ILF-CP	Age range			8.000 (2, 8)	0.018	–
	<25 y.o.	0 (0.1)	1 (0.9)			
	25–65 y.o.	0 (0.8)	6 (5.3)			
	65+ y.o.	1 (0.1)	0 (0.9)			

**TABLE 12:** Chi square analysis of independent variable effects on symptom resolution among various subgroups of PPCS patients.

		Symptom resolution?				
		
		
Treatment group		No	Yes	χ^2^ (df, N)	Asymptotic sig. (2-sided)	Fisher’s Exact Test (2-sided)
		Count (Expected)	Count (Expected)			
All PPCS	Pre-injury anxiety			7.708 (1, 59)	0.005	0.007
	No	20 (24.6)	24 (19.4)			
	Yes	13 (8.4)	2 (6.6)			
TAU	Pre-injury anxiety			4.350 (1, 26)	0.037	0.087
	No	8 (10.5)	8 (5.5)			
	Yes	9 (6.5)	1 (3.5)			

## Discussion

The prevalence of concussion cases coming to medical attention in our geographic population was 0.10%, with the majority (59.1%) referred to a neurologist. Postconcussion headache was the most frequent symptom presenting in our study in agreement with other epidemiologic investigations (Lucas et al., 2014), followed by memory disturbances and changes in cognitive processing (i.e., “brain fog”). Time from injury to treatment was comparable between TAU and TAU+ILF+CP, and extended for TAU+ILF-CP, yet subjects in both TAU+ILF+CP and TAU+ILF-CP subgroups demonstrated faster median time to first symptom improvement over TAU. Median time to first symptom resolution was comparable between TAU and TAU+ILF+CP but protracted for the TAU+ILF-CP subgroup.

We showed that TAU, TAU+ILF, TAU+ILF+CP, and TAU+ILF-CP were all significantly efficacious as standalone treatments in symptom improvement; all treatments apart from TAU+ILF-CP were also significantly efficacious in symptom resolution. Of note, the TAU+ILF+CP subgroup showed the greatest percent of patients experiencing at least one symptom improvement, at 100%. Treatment groups did not show significant changes in MoCA scores, however the TAU+ILF+CP subgroup exhibited the greatest percentage of improved cases (75%).

The ILF treatment efficacy results correspond with reports of improved clinical outcomes in TBI patients administered varied modalities of neurofeedback. Our findings also correspond with the sole previous study on use of ILF training in postconcussion, which demonstrated improved symptom scores after receiving a total of twenty 32-minute sessions of ILF neurofeedback at a frequency of three sessions per week (Carlson and Webster Ross, 2021). In our study, patients received one 50-minute session of ILF neurofeedback once to twice per week, and often with greater than 1 week between successive sessions, with a treatment goal of at least 20 sessions; despite a reduced weekly frequency, our results found similar efficacy of ILF therapy to that of Carlson and Webster Ross (2021).

The current study is, to our knowledge, the first to compare the efficacy of ILF neurofeedback training to treatment as usual in the setting of persistent post-concussion symptoms. We hypothesized that PPCS patients receiving integrative TAU+ILF therapy would show significant benefits in symptom remediation when compared to treatment as usual alone. Our hypothesis was supported by evidence that subjects receiving TAU+ILF demonstrated greater net symptom improvement over subjects receiving TAU alone. In particular, the TAU+ILF+CP subgroup exhibited more significant net symptom improvement and percent symptom improvement than patients receiving TAU, while the TAU+ILF-CP subgroup only showed non-significant trends toward greater net and percent symptom improvement. However, it was not shown that patients receiving TAU+ILF or specifically TAU+ILF+CP showed more significant net or percent resolution of symptoms.

When comparing neurofeedback session number and type, subjects receiving greater than 40 sessions of concussion protocol experienced greater net and percent symptom improvement than subjects receiving greater than 40 sessions of any other protocol; these findings were consistent at the level of 30+ sessions as well in net symptom improvement only. Additionally, patients receiving greater than 40 sessions of concussion protocol experienced greater net symptom improvement than patients receiving less than 40 sessions of concussion protocol. Inconsistent and non-significant trends were found in all other comparisons. It is likely that all forms of ILF neuromodulation provide some benefit to the patient, however we found that the concussion protocol was associated with significantly more symptom remediation over treatment as usual than other protocols, even with a minimum of 10 sessions.

We revealed via logistic regression that age, severity of traumatic brain injury, and previous diagnosis of PTSD or anxiety were significantly associated with symptom resolution in our PPCS patient group. Patients aged 25 years or less were more likely to experience symptom resolution than patients aged 65 years or more; symptoms related to mild TBI were more likely to resolve than in cases of severe TBI; patients with a concomitant diagnosis of anxiety were less likely to resolve than patients without, and patients with co-existing PTSD were more likely to resolve than patients without. These associations with age and anxiety were further supported in our chi square analyses. No significant associations were seen within the TAU group via logistic regression or chi square analysis. Therefore, there are likely multiple factors that impact clinical benefits of ILF treatment; larger cohort studies are needed.

In summary, our small, retrospective cohort study demonstrated greater clinical benefits to patients with PPCS when ILF neuromodulation was integrated with treatment as usual and particularly when ILF was used with concussion protocol, a method able to be readily duplicated by other ILF practitioners.

We agree with the descriptive term “postconcussion symptoms”; the brain has a limited repertoire in its response to significant insults and indeed these symptoms are not unique to TBI. Disruption of slow oscillatory networks are implicated, contributed to by biomechanical forces acting especially at the upper brainstem level and paralimbic structures resulting in DAI impacting restricted neuronal populations in predominantly subcortical regions, many of which contribute to critical hub activity of our task negative networks like the DMN. It is likely there are relevant slow oscillatory task-negative and task-positive networks yet to be fully identified, and the slow control system to be better characterized. ILF neuromodulation has been proposed to engage directly with and promote the regulatory activities of these slow oscillatory systems (Legarda et al., 2011); there is a hierarchy of regulatory control in the brain in which the slow control system may likely reign supreme, and its mechanism may likewise be promoted by the ILF method.

ILF neurotherapy has revolutionized the manner in which we provide neurological care to our patients with serious, debilitating central nervous system disorders. The slow control system of the brain is a phenomenon discovered almost 60 years ago; efforts in this direction of inquiry by the neuroscientific community would allow for ever-evolving non-invasive neurotherapies that offer an alternative to potential overuse of pharmacotherapeutics and illicit drugs.

### Limitations

The retrospective nature of this study posed several limitations. Our patients received individualized attention from different neurologists with individual practice habits. Not all subjects completed objective assessments (MoCA and/or MMSE) during the pre-treatment phase and even fewer completed the assessments in the post-treatment phase. A standardized postconcussion questionnaire was not used consistently before and after treatment, resulting in reliance solely on subjective data (i.e., patients’ reporting back of their symptoms to their doctors). Prospective studies following patients with mTBI have found these questionnaires and assessments offer inherent limitations and inconclusive results because of heavy reliance on patients’ subjective states and recollections, and because PCS symptoms are not unique to mTBI (McMahon et al., 2014). Some investigators found no differences in scores compared to a non-mTBI injury population (Ettenhofer and Abeles, 2009), while others did report differences (Dikmen et al., 2010). Likewise, we did not find the cognitive assessments to provide significance and found the questionnaires too uniform an approach that did not include important symptoms like anxiety, an important variable found in our study. We relied on physician records for patient testimony wherein previously self-reported symptoms were identified in follow-up assessments by their neurologist to carefully compare severity since the prior visit, providing a relatively more objective recall activity consistently performed by the neurologists in this practice.

There is selection bias inherent to this current study, as patients were not randomly assigned to receive either TAU or TAU+ILF. Often patients with mild symptoms or recent head trauma were prescribed TAU (the number of included PCS patients with TAU was twenty-four, while the number of PCS patients with TAU+ILF was one) while patients with protracted symptoms and severe head trauma were prescribed TAU+ILF. Prescribed treatments in TAU were also not consistent. Additionally, there was bias to use of the concussion protocol because there was too small a sample (*n* = 3) of non-concussion protocol patients to which we could compare our TAU+ILF+CP subgroup (*n* = 25). We therefore limited our concussion protocol group to subjects receiving at least 10 sessions of concussion protocol, while relegating subjects who received less than 10 sessions of concussion protocol (*n* = 5) to the TAU+ILF-CP group. Despite these efforts, the TAU+ILF-CP group was still small in size (*n* = 8), the majority of whom experienced beneficial effects of the concussion protocol impacting any true comparison between TAU+ILF+CP and TAU+ILF-CP.

Time between successive ILF sessions was delayed for many TAU+ILF patients due to the COVID-19 pandemic; our clinic operations were interrupted and slowed for approximately 6 months in mid-2020. These disruptions may have impacted time to improvement and time to resolution data of the ILF study subjects affected. Nonetheless, we did not see complete resolution of symptoms in the majority of our patients. Delay to treatment must be considered; time to treatment ranged from 1.7 to 3.8 years in our study. Future prospective studies would be useful in determining exact treatment windows for assessment, whether adhering to specific training protocol for a greater number of sessions confers greater resolution (we saw a trend toward resolution with 40+ sessions concussion protocol), and if earlier ILF intervention in patients with postconcussion syndrome effects more significant symptoms resolution.

### Conclusion

The current study findings support our clinical experience that ILF brain training forms a valuable part of integrative therapy in managing persistent post-concussion symptoms, a disabling condition that is growing in incidence and raising public health concerns. So-called mild TBI has been identified a risk factor for frontotemporal dementia; neuroanatomic correlates in dementia are notably similar to those discussed earlier in mTBI (Grothe et al., 2010; Aggleton et al., 2016).

A significant treatment response in terms of symptoms improvement was seen particularly with use of a concussion protocol. While symptom resolution was not a significant outcome for any modality used within the timeframe and scope of this retrospective study, a clear trend was observed wherein patients with PPCS who received TAU+ILF experienced greater resolution of symptoms [TAU+ILF+CP (52%) and TAU+ILF-CP (50%)] compared to their cohorts who received TAU (38.5%) (see Table 4). Although 67% of patients with PCS may expect to resolve their symptoms in the first three months (McMahon et al., 2014), 33% continue to experience symptoms beyond three months (PPCS). We recognize the utility of a randomized placebo-controlled prospective study wherein mTBI patients receive ILF shortly after their injury to determine if symptom resolution is more readily feasible when ILF neuromodulation is administered early.

## Data Availability Statement

The raw data supporting the conclusions of this article will be made available by the authors, without undue reservation.

## Ethics Statement

The studies involving human participants were reviewed and approved by the Community Hospital of Monterey Peninsula Institutional Review Board. Written informed consent from the participants’ legal guardian/next of kin was not required to participate in this study in accordance with the national legislation and the institutional requirements.

## Author Contributions

SL: 50% manuscript writing, submitted grant proposal for funding, physician scholarship grant awardee, initiated and managed IRB process for study approval, clinical study investigator, and literature review. CL: 100% statistical analysis and data management, 40% manuscript writing, and literature review. DM: clinical study investigator, 5% manuscript writing, and guided statistical analysis. AM-M: clinical study investigator, 5% manuscript writing, and grant support presentation. All authors contributed to the article and approved the submitted version.

## Conflict of Interest

The authors declare that the research was conducted in the absence of any commercial or financial relationships that could be construed as a potential conflict of interest.

## Publisher’s Note

All claims expressed in this article are solely those of the authors and do not necessarily represent those of their affiliated organizations, or those of the publisher, the editors and the reviewers. Any product that may be evaluated in this article, or claim that may be made by its manufacturer, is not guaranteed or endorsed by the publisher.

## Footnotes

1
https://tinyurl.com/262dfyt5
