# Supplementary material for: Use of Novel Concussion Protocol With Infralow Frequency Neuromodulation Demonstrates Significant Treatment Response in Patients With Persistent Postconcussion Symptoms, a Retrospective Study
Source: Front Hum Neurosci. 2022 May 24;16:894758. doi: 10.3389/fnhum.2022.894758 (PMC9170890; doi:10.3389/fnhum.2022.894758)
Supplement: Supplementary file 1 [file Data_Sheet_1.PDF]

Supplementary Tables

Supplementary Table 1. Excluded cases.

Exclusion Reason	n
Referred but no show to neurology	41
Study entry point occurred prior to or after designated timeframe	44
Diagnosed but not treated for PCS or history of concussion	53
Lost to follow up	9
Other *(See Methods: Subject Selection)*	2
Non-ILF neurofeedback received	1
<10 sessions of ILF neurofeedback received	9

Supplementary Table 2. Pre- vs. post-treatment analysis of Montreal Cognitive Assessments in persistent post-concussion patients.

Treatment	n	Age (mean ± SD)	Median pre-treatment score	Median post-treatment score	Significance of Difference (p)
TAU	5	48.0 ± 25.1	22	27	0.336
TAU+ILF	14	55.6 ± 25.9	23.5	24	0.148
TAU+ILF+CP	12	61.1 ± 23.5	22	23.5	0.168
TAU+ILF-CP	2	22.5 ± 10.6	26.5	26.8	0.655

Supplementary Table 3. Pre- vs. post-treatment analysis of Mini Mental Status Examinations in persistent post-concussion patients.

Treatment	n	Age (mean ± SD)	Median pre-treatment score	Median post-treatment score	Significance of Difference (p)
TAU	0	-	-	-	-
TAU+ILF	9	46.7 ± 29.8	27	26	0.526
TAU+ILF+CP	9	46.7 ± 29.8	27	26	0.526
TAU+ILF-CP	0	-	-	-	-

Supplementary Table 4. Chi square analysis of independent variable effects on MoCA results in all PPCS patients.

	MOCA Results				
	Worse Count (Expected)	Better Count (Expected)	χ2 (df, N)	Asymptotic Sig. (2-sided)	Fisher's Exact Test (2-sided)
Treatment			0.719 (2, 19)	0.698	-
TAU	2 (1.6)	3 (3.4)			
TAU+ILF+CP	3 (3.8)	9 (8.2)			
TAU+ILF-CP	1 (0.6)	1 (1.4)			
PTSD			0.223 (1, 19)	0.637	1.000
No PTSD	4 (4.4)	10 (9.6)			
PTSD	2 (1.6)	3 (3.4)			
Pre-injury Depression			1.032 (1, 19)	0.310	1.000
No	6 (5.4)	11 (11.6)			
Yes	0 (0.6)	2 (1.4)			
Pre-injury Anxiety			0.101 (1, 19)	0.750	1.000
No	5 (4.7)	10 (10.3)			
Yes	1 (1.3)	3 (2.7)			
Age Range			0.652 (2, 19)	0.722	-
<25 y.o.	1 (1.3)	3 (2.7)			
25-65 y.o.	3 (2.2)	4 (4.8)			
65+ y.o.	2 (2.5)	6 (5.5)			
Gender			0.693 (1, 19)	0.405	0.628
Male	4 (3.2)	6 (6.8)			
Female	2 (2.8)	7 (6.2)			
History of Multiple Concussions			3.132 (1, 19)	0.077	0.128
No	6 (4.4)	8 (9.6)			
Yes	0 (1.6)	5 (3.4)			
TBI Severity			0.263 (2, 19)	0.877	-
Mild	3 (3.5)	8 (7.5)			
Moderate	1 (0.9)	2 (2.1)			
Severe	2 (1.6)	3 (3.4)			
History of or Current Substance Dependence			4.843 (1, 19)	0.028	0.088
No	4 (5.4)	13 (11.6)			
Yes	2 (0.6)	0 (1.4)			

Supplementary Table 5. Comparative analysis of TAU versus TAU+ILF and subgroups in the resolution of PPCS symptoms.

Groups	n	Median (Q1-Q3) Δ Symptoms	Mean Rank	Sum of Ranks	U	Significance (p)	Trend
TAU	26	0 (0-1)	26.81	697.00	346.00	0.161	ILF > TAU
TAU+ILF	33	1 (0-2)	32.52	1073.00			
TAU	26	0 (0-1)	23.56	612.50	261.50	0.183	ILF > TAU
TAU+ILF+CP	25	1 (0-2)	28.54	713.50			
TAU	26	0 (0-1)	16.75	435.50	84.50	0.363	ILF > TAU
TAU+ILF-CP	8	0.5 (0-2)	19.94	159.50			
TAU+ILF+CP	25	1 (0-2)	17.14	428.50	96.50	0.875	+CP > -CP
TAU+ILF-CP	8	0.5 (0-2)	16.56	132.50			

A Mann-Whitney U test assessed differences in resolved symptom quantities between treatment groups. The following comparisons were made: TAU vs. TAU+ILF, TAU vs. TAU+ILF+CP, TAU vs. TAU+ILF-CP, and TAU+ILF+CP vs. TAU+ILF-CP. Statistically significant values ($p<0.05$) indicate tendency for greater number of symptoms improved or resolved in one of the compared groups; these are highlighted in red. The "Trend" column highlights the direction of greater value in the comparison, e.g., whether the overall number of improved or resolved symptoms was greater for the TAU+ILF (sub)group or TAU. The TAU+ILF group and its subgroups are abbreviated to "ILF" in the Trend column.

Supplementary Table 6. Comparative analysis of TAU versus TAU+ILF and subgroups in the percent resolution of PPCS symptoms.

Groups	n	Median (Q1-Q3) % Symptom Change	Mean Rank	Sum of Ranks	U	p	Trend
TAU	26	0.00% (0.00-38.3%)	28.54	742.00	391.00	0.523	ILF > TAU
TAU+ILF	33	14.3% (0.00-25.0%)	31.15	1028.00			
TAU	26	0.00% (0.00-38.3%)	24.87	646.50	295.50	0.538	ILF > TAU
TAU+ILF+CP	25	14.3% (0.00-25.0%)	27.18	679.50			
TAU	26	0.00% (0.00-38.3%)	17.17	446.50	95.50	0.692	ILF > TAU
TAU+ILF-CP	8	12.5% (0.00-27.1%)	18.56	148.50			
TAU+ILF+CP	25	14.3% (0.00-25.0%)	16.86	421.50	96.50	0.876	-CP > +CP
TAU+ILF-CP	8	12.5% (0.00-27.1%)	17.44	139.50			

A Mann-Whitney U test assessed differences in percentage of symptoms resolved between treatment groups. The following comparisons were made: TAU vs. TAU+ILF, TAU vs. TAU+ILF+CP, TAU vs. TAU+ILF-CP, and TAU+ILF+CP vs. TAU+ILF-CP. Statistically significant values ($p<0.05$) indicate tendency for greater percent symptom improvement or resolution in one of the compared groups; these are highlighted in red. The "Trend" column highlights the direction of greater value in the comparison, e.g., whether the overall percentage of improved or resolved symptoms was greater for TAU or TAU+ILF. The TAU+ILF group and its subgroups are abbreviated to "ILF" in the Trend column.

Supplementary Table 7. Comparative analysis of number and type of neurofeedback sessions on symptom resolution status.

Protocol Comparisons	n	Median (Q1-Q3) Δ Symptoms	Mean Rank	Sum of Ranks	U	p	Trend
1-9 CP	5	1 (0-2)	16.20	81.00	59.00	0.836	<10 > 10+
10+ CP	25	1 (0-2)	15.36	384.00			
1-19 CP	20	1 (0-3)	16.05	321.00	89.00	0.607	<20 > 20+
20+ CP	10	0.5 (0-1)	14.40	144.00			
1-29 CP	26	1 (0-2)	15.46	402.00	51.00	0.948	30+ > <30
30+ CP	4	0.5 (0-2.25)	15.75	63.00			
1-39 CP	27	1 (0-2)	15.17	409.50	31.50	0.508	40+ > <40
40+ CP	3	1 (0.5-3.5)	18.50	55.50			
20+ Any	11	1 (0-1.5)	11.68	128.50	47.50	0.574	Any > CP
20+ CP	10	0.5 (0-1)	10.25	102.50			
30+ Any	11	1 (0-1)	7.86	86.50	20.50	0.831	CP > Any
30+ CP	4	0.5 (0-2.25)	8.38	33.50			
40+ Any	6	0.5 (0-1)	4.50	27.00	6.00	0.396	CP > Any
40+ CP	3	1 (0.5-3.5)	6.00	18.00			

The number and type of neurofeedback sessions were analyzed for discrepancies in net symptom resolution. Analysis was conducted via Mann Whitney U test.

Supplementary Table 8. Comparative analysis of number and type of neurofeedback sessions on percent symptom resolution.

Protocol Comparisons	n	Median (Q1-Q3) % Δ Symptoms	Mean Rank	Sum of Ranks	U	p	Trend
1-9 CP	5	25.0% (0.00-33.3%)	17.70	88.50	51.50	0.518	<10 > 10+
10+ CP	25	14.3% (0.00-25.0%)	15.06	376.50			
1-19 CP	20	14.3% (0.00-61.7%)	16.08	321.50	88.50	0.593	<20 > 20+
20+ CP	10	8.33% (0.00-22.9%)	14.35	143.50			
1-29 CP	26	14.3% (0.00-31.3%)	15.54	404.00	51.00	0.949	<30 > 30+
30+ CP	4	8.33% (0.00-33.9%)	15.25	61.00			
1-39 CP	27	14.3% (0.00-29.2%)	15.24	411.50	33.50	0.609	40+ > <40
40+ CP	3	16.6% (8.33-51.2%)	17.83	53.50			
20+ Any	11	14.3% (0.00-25.0%)	11.55	127.00	49.00	0.658	Any > CP
20+ CP	10	8.33% (0.00-22.9%)	10.40	104.00			
30+ Any	11	14.3% (0.00-16.7%)	7.82	86.00	20.00	0.782	CP > Any
30+ CP	4	8.33% (0.00-33.9%)	8.50	34.00			
40+ Any	6	7.14% (0.00-16.1%)	4.42	26.50	5.50	0.336	CP > Any
40+ CP	3	16.6% (8.33-51.2%)	6.17	18.50			

The number and type of neurofeedback sessions were analyzed for discrepancies in percent symptom resolution. Analysis was conducted via Mann Whitney U test.

Supplementary Table 9. Binary logistic regression of possible confounding factors on persistent post-concussion symptom improvement (n=59).

		Sig.	Odds Ratio (OR)	95% C.I. for OR	
				Lower	Upper
Independent Variable	Treatment (ref. TAU+ILF-CP)	1.000			
	TAU	0.996	4.308×10^{6}	0.000	-
	TAU+ILF+CP	0.996	4.572×10^{14}	0.000	-
	PTSD (ref. Yes)	0.997	0.000	0.000	-
	Depression (ref. Yes)	0.995	0.000	0.000	-
	Anxiety (ref. Yes)	0.996	4.827×10^{13}	0.000	-
	Age Range (ref. 65+ y.o.)	0.328			
	<25 y.o.	1.000	42.788	0.000	-
	25-65 y.o.	0.136	11.234	0.469	269.184
	Gender (ref. Female)	0.919	1.312	0.007	243.015
	History of Multiple Concussions (ref. Yes)	0.996	3.681×10^{13}	0.000	-
	TBI Severity (ref. Severe)	1.000			
	Mild	0.996	0.000	0.000	-
	Moderate	0.999	0.000	0.000	-
	History of/Present Substance Misuse (ref. Yes)	0.997	1.121×10^{7}	0.000	-
	Constant	0.998	8.580×10^{13}		
Model Statistics	Percentage of Accuracy of Classification	96.6%			
	Model Significance	0.016			
	Nagelkerke R Square	0.780			
	Hosmer and Lemeshow Test Sig.	0.897			

A binary logistic regression assessed for variable effects on symptom resolution among persistent post-concussion patients (n=59). The dependent variable was set to occurrence of symptom resolution (0=no, 1= yes).

Supplementary Table 10. Binary logistic regression of possible confounding factors on PPCS symptom resolution among TAU patients only (n=26).

		Sig.	Odds Ratio (OR)	95% C.I. for OR	
				Lower	Upper
Independent Variable	TAU or TAU+ILF (ref. TAU+ILF)	-	-	-	-
	PTSD (ref. Yes)	1.000	2.011	0.000	-
	Depression (ref. Yes)	0.999	0.000	0.000	-
	Anxiety (ref. Yes)	0.998	5.468x10^{16}	0.000	-
	Age Range (ref. 65+ y.o.)	1.000			
	<25 y.o.	0.999	3.934x10^{8}	0.000	-
	25-65 y.o.	0.999	0.000	0.000	-
	Gender (ref. Female)	0.890	0.783	0.024	25.160
	History of Multiple Concussions (ref. Yes)	0.999	9.687x10^{8}	0.000	-
	TBI Severity (ref. Severe)	0.991			
	Mild	0.999	2.183x10^{8}	0.000	-
	Moderate	0.894	0.718	0.005	93.689
	History of/Present Substance Misuse (ref. Yes)	0.904	1.280	0.023	71.144
	Constant	0.998	0.000		
Model Statistics	Percentage of Accuracy of Classification	80.8%			
	Model Significance	0.077			
	Nagelkerke R Square	0.659			
	Hosmer and Lemeshow Test Sig.	0.782			

A binary logistic regression assessed for variable effects on symptom resolution among persistent post-concussion patients receiving treatment as usual only (n=26). The dependent variable was set to occurrence of symptom resolution (0=no, 1= yes).

Supplementary Table 11. Chi Square Analysis of Independent Variable Effects on Symptom Improvement in PPCS patients.

	Symptom Improvement? No Count (Expected)	Yes Count (Expected)	$\chi 2$ (df, N)	Asymptotic Sig. (2-sided)	Fisher's Exact Test (2-sided)
Treatment			4.083 (2, 59)	0.130	-
TAU	4 (2.2)	22 (23.8)			
TAU+ILF+CP	0 (2.1)	25 (22.9)			
TAU+ILF-CP	1 (0.7)	7 (7.3)			
PTSD			1.252 (1, 59)	0.263	0.572
No PTSD	5 (4.1)	43 (43.9)			
PTSD	0 (0.9)	11 (10.1)			
Pre-injury Depression			0.459 (1, 59)	0.498	0.606
No	3 (3.6)	40 (39.4)			
Yes	2 (1.4)	14 (14.6)			
Pre-injury Anxiety			3.445 (1, 59)	0.063	0.099
No	2 (3.7)	42 (40.3)			
Yes	3 (1.3)	12 (13.7)			
Age Range			7.062 (2, 59)	0.029	-
<25 y.o.	0 (0.8)	9 (8.2)			
25-65 y.o.	1 (2.8)	32 (30.2)			
65+ y.o.	4 (1.4)	13 (15.6)			
Gender			0.257 (1, 59)	0.612	0.671
Male	3 (2.5)	26 (26.5)			
Female	2 (2.5)	28 (27.5)			
History of Multiple Concussions			2.988 (1, 59)	0.084	0.118
No	2 (3.6)	41 (39.4)			
Yes	3 (1.4)	13 (14.6)			
TBI Severity			3.490 (2, 59)	0.175	-
Mild	5 (3.1)	31 (32.9)			
Moderate	0 (0.6)	7 (6.4)			
Severe	0 (1.4)	16 (14.6)			
History of or Current Substance Dependence			0.346 (1, 59)	0.557	0.481
No	4 (4.4)	48 (47.6)			
Yes	1 (0.6)	6 (6.4)			

Supplementary Table 12. Chi Square Analysis of Independent Variable Effects on Symptom Improvement in TAU.

	Symptom Improvement?				
	No Count (Expected)	Yes Count (Expected)	χ2 (df, N)	Asymptotic Sig. (2-sided)	Fisher's Exact Test (2-sided)
PTSD			1.126 (1, 26)	0.289	0.555
No PTSD	4 (3.2)	17 (17.8)			
PTSD	0 (0.8)	5 (4.2)			
Pre-injury Depression			0.074 (1, 26)	0.786	1.000
No	3 (2.8)	15 (15.2)			
Yes	1 (1.2)	7 (6.8)			
Pre-injury Anxiety			0.266 (1, 26)	0.606	0.625
No	2 (2.5)	14 (13.5)			
Yes	2 (1.5)	8 (8.5)			
Age Range			3.503 (2, 26)	0.173	-
<25 y.o.	0 (0.5)	3 (2.5)			
25-65 y.o.	1 (2.2)	13 (11.8)			
65+ y.o.	3 (1.4)	6 (7.6)			
Gender			0.028 (1, 26)	0.867	1.000
Male	2 (2.2)	12 (11.8)			
Female	2 (1.8)	10 (10.2)			
History of Multiple Concussions			4.342 (1, 26)	0.037	0.072
No	1 (2.8)	17 (15.2)			
Yes	3 (1.2)	5 (6.8)			
TBI Severity			2.101 (2, 26)	0.350	-
Mild	4 (2.8)	14 (15.2)			
Moderate	0 (0.3)	2 (1.7)			
Severe	0 (0.9)	6 (5.1)			
History of or Current Substance Dependence			1.126 (1, 26)	0.289	0.555
No	4 (3.2)	17 (17.8)			
Yes	0 (0.8)	5 (4.2)			

Supplementary Table 13. Chi Square Analysis of Independent Variable Effects on Symptom Improvement in TAU+ILF.

	Symptom Improvement? No Count (Expected)	Symptom Improvement? Yes Count (Expected)	χ2 (df, N)	Asymptotic Sig. (2-sided)	Fisher's Exact Test (2-sided)
Treatment			3.223 (1, 33)	0.073	0.242
TAU+ILF+CP	0 (0.8)	25 (24.2)			
TAU+ILF-CP	1 (0.2)	7 (7.8)			
PTSD			0.229 (1, 33)	0.632	1.000
No PTSD	1 (0.8)	26 (26.2)			
PTSD	0 (0.2)	6 (5.8)			
Pre-injury Depression			3.223 (1, 33)	0.073	0.242
No	0 (0.8)	25 (24.2)			
Yes	1 (0.2)	7 (7.8)			
Pre-injury Anxiety			5.775 (1, 33)	0.016	0.152
No	0 (0.8)	28 (27.2)			
Yes	1 (0.2)	4 (4.8)			
Age Range			3.223 (2, 33)	0.200	-
<25 y.o.	0 (0.2)	6 (5.8)			
25-65 y.o.	0 (0.6)	19 (18.4)			
65+ y.o.	1 (0.2)	7 (7.8)			
Gender			1.237 (1, 33)	0.266	0.455
Male	1 (0.5)	14 (14.5)			
Female	0 (0.5)	18 (17.5)			
History of Multiple Concussions			0.330 (1, 33)	0.566	1.000
No	1 (0.8)	24 (24.2)			
Yes	0 (0.2)	8 (7.8)			
TBI Severity			0.859 (2, 33)	0.651	-
Mild	1 (0.5)	17 (17.5)			
Moderate	0 (0.2)	5 (4.8)			
Severe	0 (0.3)	10 (9.7)			
History of or Current Substance Dependence			15.984 (1, 33)	<0.001	0.061
No	0 (0.9)	31 (30.1)			
Yes	1 (0.1)	1 (1.9)			

Supplementary Table 14. Chi Square Analysis of Independent Variable Effects on Symptom Improvement in TAU+ILF-CP.

	Symptom Improvement?				
	No Count (Expected)	Yes Count (Expected)	χ2 (df, N)	Asymptotic Sig. (2-sided)	Fisher's Exact Test (2-sided)
PTSD			0.686 (1, 8)	0.408	1.000
No PTSD	1 (0.6)	4 (4.4)			
PTSD	0 (0.4)	3 (2.6)			
Pre-injury Depression			1.905 (1, 8)	0.168	0.375
No	0 (0.6)	5 (4.4)			
Yes	1 (0.4)	2 (2.6)			
Pre-injury Anxiety			1.905 (1, 8)	0.168	0.375
No	0 (0.6)	5 (4.4)			
Yes	1 (0.4)	2 (2.6)			
Age Range			8.000 (2, 8)	0.018	-
<25 y.o.	0 (0.1)	1 (0.9)			
25-65 y.o.	0 (0.8)	6 (5.3)			
65+ y.o.	1 (0.1)	0 (0.9)			
Gender			1.905 (1, 8)	0.168	0.375
Male	1 (0.4)	2 (2.6)			
Female	0 (0.6)	5 (4.4)			
History of Multiple Concussions			0.686 (1, 8)	0.408	1.000
No	1 (0.6)	4 (4.4)			
Yes	0 (0.4)	3 (2.6)			
TBI Severity			0.163 (1, 8)	0.686	1.000
Mild	1 (0.9)	6 (6.1)			
Moderate	-	-			
Severe	0 (0.1)	1 (0.9)			
History of or Current Substance Dependence			3.429 (1, 8)	0.064	0.250
No	0 (0.8)	6 (5.3)			
Yes	1 (0.3)	1 (1.8)			

Supplementary Table 15. Chi Square Analysis of Independent Variable Effects on Symptom Resolution in PPCS patients.

	Symptom Resolution?				
	No<br>Count (Expected)	Yes<br>Count (Expected)	$\chi 2$ (df, N)	Asymptotic Sig.<br>(2-sided)	Fisher's Exact<br>Test (2-sided)
Treatment			1.695 (2, 59)	0.429	-
TAU	17 (14.5)	9 (11.5)			
TAU+ILF+CP	12 (14.0)	13 (11.0)			
TAU+ILF-CP	4 (4.5)	4 (3.5)			
PTSD			0.011 (1, 59)	0.918	1.000
No PTSD	27 (26.8)	21 (21.2)			
PTSD	6 (6.2)	5 (4.8)			
Pre-injury Depression			3.238 (1, 59)	0.072	0.085
No	21 (24.1)	22 (18.9)			
Yes	12 (8.9)	4 (7.1)			
Pre-injury Anxiety			7.708 (1, 59)	0.005	0.007
No	20 (24.6)	24 (19.4)			
Yes	13 (8.4)	2 (6.6)			
Age Range			5.002 (2, 59)	0.082	-
<25 y.o.	2 (5.0)	7 (4.0)			
25-65 y.o.	21 (18.5)	12 (14.5)			
65+ y.o.	10 (9.5)	7 (7.5)			
Gender			2.126 (1, 59)	0.145	0.192
Male	19 (16.2)	10 (12.8)			
Female	14 (16.8)	16 (13.2)			
History of Multiple Concussions			0.384 (1, 59)	0.535	0.571
No	23 (24.1)	20 (18.9)			
Yes	10 (8.9)	6 (7.1)			
TBI Severity			4.632 (2, 59)	0.099	-
Mild	19 (20.1)	17 (15.9)			
Moderate	2 (3.9)	5 (3.1)			
Severe	12 (8.9)	4 (7.1)			
History of or Current Substance Dependence			0.774 (1, 59)	0.379	0.449
No	28 (29.1)	24 (22.9)			
Yes	5 (3.9)	2 (3.1)			

Supplementary Table 16. Chi Square Analysis of Independent Variable Effects on Symptom Resolution in TAU.

	Symptom Resolution? No Count (Expected)	Yes Count (Expected)	χ2 (df, N)	Asymptotic Sig. (2-sided)	Fisher's Exact Test (2-sided)
PTSD			0.584 (1, 26)	0.445	0.628
No PTSD	13 (13.7)	8 (7.3)			
PTSD	4 (3.3)	1 (1.7)			
Pre-injury Depression			0.472 (1, 26)	0.492	0.667
No	11 (11.8)	7 (6.2)			
Yes	6 (5.2)	2 (2.8)			
Pre-injury Anxiety			4.350 (1, 26)	0.037	0.087
No	8 (10.5)	8 (5.5)			
Yes	9 (6.5)	1 (3.5)			
Age Range			1.594 (2, 26)	0.451	-
<25 y.o.	1 (2.0)	2 (1.0)			
25-65 y.o.	10 (9.2)	4 (4.8)			
65+ y.o.	6 (5.9)	3 (3.1)			
Gender			0.016 (1, 26)	0.899	1.000
Male	9 (9.2)	5 (4.8)			
Female	8 (7.8)	4 (4.2)			
History of Multiple Concussions			2.497 (1, 26)	0.114	0.190
No	10 (11.8)	8 (6.2)			
Yes	7 (5.2)	1 (2.8)			
TBI Severity			1.208 (2, 26)	0.547	-
Mild	11 (11.8)	7 (6.2)			
Moderate	1 (1.3)	1 (0.7)			
Severe	5 (3.9)	1 (2.1)			
History of or Current Substance Dependence			0.079 (1, 26)	0.778	1.000
No	14 (13.7)	7 (7.3)			
Yes	3 (3.3)	2 (1.7)			

Supplementary Table 17. Chi Square Analysis of Independent Variable Effects on Symptom Resolution in TAU+ILF.

	Symptom Resolution? No Count (Expected)	Symptom Resolution? Yes Count (Expected)	χ2 (df, N)	Asymptotic Sig. (2-sided)	Fisher's Exact Test (2-sided)
Treatment			0.010 (1, 33)	0.922	1.000
TAU+ILF+CP	12 (12.1)	13 (12.9)			
TAU+ILF-CP	4 (3.9)	4 (4.1)			
PTSD			0.674 (1, 33)	0.412	0.656
No PTSD	14 (13.1)	13 (13.9)			
PTSD	2 (2.9)	4 (3.1)			
Pre-injury Depression			2.972 (1, 33)	0.085	0.118
No	10 (12.1)	15 (12.9)			
Yes	6 (3.9)	2 (4.1)			
Pre-injury Anxiety			2.343 (1, 33)	0.126	0.175
No	12 (13.6)	16 (14.4)			
Yes	4 (2.4)	1 (2.6)			
Age Range			3.113 (2, 33)	0.211	-
<25 y.o.	1 (2.9)	5 (3.1)			
25-65 y.o.	11 (9.2)	8 (9.8)			
65+ y.o.	4 (3.9)	4 (4.1)			
Gender			3.640 (1, 33)	0.056	0.084
Male	10 (7.3)	5 (7.7)			
Female	6 (8.7)	12 (9.3)			
History of Multiple Concussions			0.510 (1, 33)	0.475	0.688
No	13 (12.1)	12 (12.9)			
Yes	3 (3.9)	5 (4.1)			
TBI Severity			3.595 (2, 33)	0.166	-
Mild	8 (8.7)	10 (9.3)			
Moderate	1 (2.4)	4 (2.6)			
Severe	7 (4.8)	3 (5.2)			
History of or Current Substance Dependence			2.262 (1, 33)	0.133	0.227
No	14 (15.0)	17 (16.0)			
Yes	2 (1.0)	0 (1.0)			

Supplementary Table 18. Chi Square Analysis of Independent Variable Effects on Symptom Resolution in TAU+ILF+CP.

	Symptom Resolution? No Count (Expected)	Symptom Resolution? Yes Count (Expected)	χ2 (df, N)	Asymptotic Sig. (2-sided)	Fisher's Exact Test (2-sided)
PTSD			0.294 (1, 25)	0.588	1.000
No PTSD	11 (10.6)	11 (11.4)			
PTSD	1 (1.4)	2 (1.6)			
Pre-injury Depression			2.564 (1, 25)	0.109	0.160
No	8 (9.6)	12 (10.4)			
Yes	4 (2.4)	1 (2.6)			
Pre-injury Anxiety			2.355 (1, 25)	0.125	0.220
No	10 (11.0)	13 (12.0)			
Yes	2 (1.0)	0 (1.0)			
Age Range			2.599 (2, 25)	0.273	-
<25 y.o.	1 (2.4)	4 (2.6)			
25-65 y.o.	8 (6.2)	5 (6.8)			
65+ y.o.	3 (3.4)	4 (3.6)			
Gender			0.987 (1, 25)	0.320	0.434
Male	7 (5.8)	5 (6.2)			
Female	5 (6.2)	8 (6.8)			
History of Multiple Concussions			1.963 (1, 25)	0.161	0.322
No	11 (9.6)	9 (10.4)			
Yes	1 (2.4)	4 (2.6)			
TBI Severity			2.855 (2, 25)	0.240	-
Mild	5 (5.3)	6 (5.7)			
Moderate	1 (2.4)	4 (2.6)			
Severe	6 (4.3)	3 (4.7)			
History of or Current Substance Dependence			-	-	-
No	12 (12.0)	13 (13.0)			
Yes	-	-			

Supplementary Table 19. Chi Square Analysis of Independent Variable Effects on Symptom Resolution in TAU+ILF-CP.

	Symptom Resolution?				
	No<br>Count (Expected)	Yes<br>Count (Expected)	$\chi2$ (df, N)	Asymptotic Sig. (2-sided)	Fisher's Exact Test (2-sided)
PTSD			0.533 (1, 8)	0.465	1.000
No PTSD	3 (2.5)	2 (2.5)			
PTSD	1 (1.5)	2 (1.5)			
Pre-injury Depression			0.533 (1, 8)	0.465	1.000
No	2 (2.5)	3 (2.5)			
Yes	2 (1.5)	1 (1.5)			
Pre-injury Anxiety			0.533 (1, 8)	0.465	1.000
No	2 (2.5)	3 (2.5)			
Yes	2 (1.5)	1 (1.5)			
Age Range			2.000 (2, 8)	0.368	-
<25 y.o.	0 (0.5)	1 (0.5)			
25-65 y.o.	3 (3.0)	3 (3.0)			
65+ y.o.	1 (0.5)	0 (0.5)			
Gender			4.800 (1, 8)	0.028	0.143
Male	3 (1.5)	0 (1.5)			
Female	1 (2.5)	4 (2.5)			
History of Multiple Concussions			0.533 (1, 8)	0.465	1.000
No	2 (2.5)	3 (2.5)			
Yes	2 (1.5)	1 (1.5)			
TBI Severity			1.143 (1, 8)	0.285	1.000
Mild	3 (3.5)	4 (3.5)			
Moderate	-	-			
Severe	1 (0.5)	0 (0.5)			
History of or Current Substance Dependence			2.667 (1, 8)	0.102	0.429
No	2 (3.0)	4 (3.0)			
Yes	2 (1.0)	0 (1.0)			
